# Neural correlates of adaptive social responses to real-life frustrating situations: a functional MRI study

**DOI:** 10.1186/1471-2202-14-29

**Published:** 2013-03-13

**Authors:** Atsushi Sekiguchi, Motoaki Sugiura, Satoru Yokoyama, Yuko Sassa, Kaoru Horie, Shigeru Sato, Ryuta Kawashima

**Affiliations:** 1 Division of Medical Neuroimage Analysis, Department of Community Medical Supports, Tohoku Medical Megabank Organization, Tohoku University, Seiryo-machi, Aoba-ku, Sendai 980-8575, Japan; 2 Department of Functional Brain Imaging, IDAC, Tohoku University, Sendai, Japan; 3 International Research Institute of Disaster Science, Tohoku University, Sendai, Japan; 4 Division of Developmental Cognitive Neuroscience, IDAC, Tohoku University, Sendai, Japan; 5 The Graduate School of Language and Cultures, Nagoya University, Nagoya, Japan; 6 Professor Emeritus, Tohoku University, Sendai, Japan

**Keywords:** Adaptive social behavior, Causal attribution, Anterior temporal lobe, Integration

## Abstract

**Background:**

Frustrating situations are encountered daily, and it is necessary to respond in an adaptive fashion. A psychological definition states that adaptive social behaviors are “self-performing” and “contain a solution.” The present study investigated the neural correlates of adaptive social responses to frustrating situations by assessing the dimension of causal attribution. Based on attribution theory, internal causality refers to one’s aptitudes that cause natural responses in real-life situations, whereas external causality refers to environmental factors, such as experimental conditions, causing such responses. To investigate the issue, we developed a novel approach that assesses causal attribution under experimental conditions. During fMRI scanning, subjects were required to engage in virtual frustrating situations and play the role of protagonists by verbalizing social responses, which were socially adaptive or non-adaptive. After fMRI scanning, the subjects reported their causal attribution index of the psychological reaction to the experimental condition. We performed a correlation analysis between the causal attribution index and brain activity. We hypothesized that the brain region whose activation would have a positive and negative correlation with the self-reported index of the causal attributions would be regarded as neural correlates of internal and external causal attribution of social responses, respectively.

**Results:**

We found a significant negative correlation between external causal attribution and neural responses in the right anterior temporal lobe for adaptive social behaviors.

**Conclusion:**

This region is involved in the integration of emotional and social information. These results suggest that, particularly in adaptive social behavior, the social demands of frustrating situations, which involve external causality, may be integrated by a neural response in the right anterior temporal lobe.

## Background

**Figure 1:**
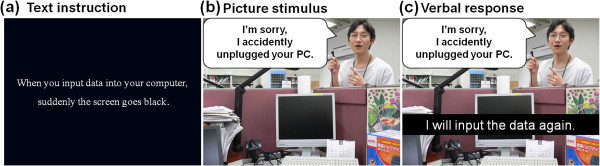
**Example of a frustrating situation (a stimulus sequence).** (**a**) Background explanation of the picture stimulus. (**b**) An example of the picture stimulus representing a frustrating situation. (**c**) An example of a verbal response as an adaptive social behavior.

We often encounter frustrating situations in our social lives. The following situation is an example: You are inputting data into your computer. Suddenly, the screen goes black, and soon after, your colleague holds up a plug and says, “I’m sorry; I accidently unplugged your PC” (Figure 1a, b). Rosenzweig created a model for the verbal responses to such frustrating situations, which was implemented in a widely used questionnaire, the “Picture-Frustration Study” (PF Study) [1,2]. In Rosenzweig’s model, verbal responses to the frustrating situation are classified by two factors: the direction and type of aggression [1,2]. The direction of aggression involves requests towards self, another person, and nothing, and the types of aggression include attention to the frustrating event itself, to a cause of the frustrating situation, and to a solution to the frustrating situation.

Social adaptation is a crucial aspect of such social responses. Also in Rosenzweig’s model, social adaptation of verbal responses is assessed by the Group Conformity Rating (GCR), which represents the degree to which these responses conform to common sense. However, the definition of social adaptation in this model was quite ambiguous, because “common sense” may differ across cultures. One clear definition of adaptation, drawn from coping theory [3,4], is the process used to manage environmental demands and to solve the problem that caused the frustrating situation without putting a burden on the environment. Corresponding to this definition [3,4], an adaptive response in Rosenzweig’s model is one with a request to self (self-performing) that contains a solution [1]. In the above-mentioned example of a frustrating situation, verbal responses such as “You need to re-input the data” (other-performing) or “I should have saved the data” (no solution) are non-adaptive because they do not manage the environmental demand to finish the data input (containing a solution), nor do they avoid putting a burden on the environment (self-performing). In this case, a verbal response such as “I will input the data again” is an adaptive social response because it suggests self-performing behavior (i.e., action by one’s self) and manages the environmental demands with a solution (i.e., to input the data again).

Causal attribution is another key dimension of adaptive social responses. Attribution theory suggests that causal attribution in social behavior has an external–internal dimension [5]. Internal causality identifies one’s aptitude as causing natural responses, whereas external causality identifies environmental factors as causing less natural responses [5]. Here, we hypothesized that natural and less natural responses were related to internal and external causal attributions, respectively. Assessment of the external–internal dimension of the causal attributions in adaptive social responses is important in areas of clinical psychology such as motivational interviewing [6], where the goal is to change clients’ behavior [7,8]. This technique has been used alter smoking behavior [9], alcohol consumption [10], and drug addiction [8], as well as to promote diet/exercise therapy for patients with obesity [11] and/or diabetes [12]. The goal of a motivational interview with such clients is to evoke an internal causal attribution to change counterproductive to adaptive behavior [6]. Thus, an objective assessment tool for the external–internal dimension of the causal attribution underlying adaptive social responses would be useful in the clinical setting. Recent development of a visual cortex decoding device using neuroimaging techniques, [13] indicates that neuroimaging studies have the potential to provide objective assessment tools for the clinical setting.

Although the dimension of causal attribution is a critical aspect of social adaptation, the neural correlates of causal attributions in adaptive social responses are not well understood. Several neuroimaging studies have investigated social adaptation in terms of moral cognition and social norms (for review, see [14,15]). In paticular, Moll et al. [16] reported that neural activity in the fronto–temporal areas was involved in moral judgment and moral sensitivity [17]. Berthoz et al. [18] demonstrated that the medial frontal and anterior temporal regions were activated during transgression of a social norm. In terms of causal attributions, Spitzer et al. [5] investigated the neural correlates of social norm compliance induced by punishment by others, which is thought to be associated with external causal attributions, and suggested that the bilateral orbitofrontal cortices and right dorsolateral prefrontal cortex were involved in compliance with social norms [19]. Recent fMRI studies have focused on the neural correlates of causal attributions made in social situations. Blackwood et al. [20] reported pre-motor cortex and cerebellar involvement in internal causal attributions, and Seidel et al. [21] showed that, compared with external attribution, internal causal attribution induced activation in the right temporoparietal junction (TPJ), and external attribution induced activation in the left TPJ and precuneus. Previous neuroimaging studies have investigated the neural correlates of either social adaptation or causal attribution in social situations; however, no studies have examined causal attribution in relation to social adaptation.

**Figure 2:**
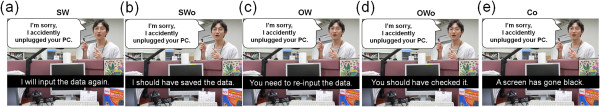
**Example of a stimulus set.** (**a**) The SW response describes the subject’s own behavior in the near future. (**b**) The SWo response describes the subject’s own behavior in the past. (**c**) The OW response describes another’s behavior in the near future. (**d**) The OWo response describes another’s behavior in the past. (**e**) The Co (control) condition only describes the situation.

The present study aimed to identify the neural correlates underlying adaptive social responses to a frustrating situation by assessing causal attributions. To this end, we created an “acting task” that applied frustrating situations to Rosenzweig’s model. We also used a parametric index of the self-reported social behavior causal attributions that was based on the work of Blackwood et al. and Seidel et al. [20,21]. We refer to this index as a “causality score,” and applied it to the acting task to assess causal attributions related to the social responses exhibited under the experimental conditions. During the acting task, each subject was presented with a virtual frustrating situation and was asked to play the role of the protagonist by verbalizing social responses (Figure 1) that were either socially adaptive or non-adaptive (Table 1, Figure 2) while undergoing functional magnetic resonance imaging (fMRI). Following fMRI scans, subjects were asked to evaluate their own causality scores, which indicated the likelihood that they would have responded to the social situation in the same way as the protagonist in the virtual frustrating situation (i.e., internal causal attribution). The causality score was used as an index of the internal causal attribution of the described social response. We assumed that causality scores would differ across trials during the acting task. We hypothesized that the brain region whose activation would have a positive and negative correlation with the self-reported index of the causal attributions would be regarded as neural correlates of internal and external causal attribution of social responses, respectively. In other words, the brain region whose neural activity during verbalization of a more natural response (i.e., internal causality) would be associated with higher causality scores than those during verbalization of a less natural social response (i.e., external causality).

**Table 1:** Examples of social responses

		**Adaptive**	**Non-adaptive**		
**Examples of verbal responses**		**I will input the data again**	**I should have saved the data**	**You need to re-input the data**	**You should have checked it**
Factors of adaptiveness	Self-performing	+	+	-	-
	Contains solution	+	-	+	-

For behavioral results, we predicted that the causality scores of the adaptive social responses would correlate significantly with the results of the psychological questionnaire scales associated with character and aggressive behavior because responses to frustrating situations are affected by individual levels of social cooperation and correspond to features of aggressive behavior [1]. In particular, we predicted that the causality scores for adaptive verbal responses would correlate with the cooperativeness (C) subscale of the Temperament and Character Inventory (TCI) [22] and the anger control subscale of the State–Trait Anger Expression Inventory (STAXI) [23], which evaluates socially cooperative characteristics and self-control over angry behavior, respectively.

We predicted that the fMRI results would show involvement of the premotor cortex, cerebellum, and right TPJ in natural social responses because these areas play a role in internal causal attribution in social situations [20,21]. We expected the left TPJ and precuneus to be associated with less natural social responses because activity in these regions is associated with external causal attribution [20,21]. Furthermore, the processing underpinning social adaptation involves a wide variety of brain regions, including the medial and lateral prefrontal cortices, anterior and posterior superior temporal sulcus, and TPJ [14,15]. Thus, we predicted that the right and left TPJ would be involved in the internal and external causal attributions made about adaptive social responses to frustrating situations, respectively.

## Methods

### Subjects

In total, 40 healthy volunteers (eight females, 32 males; mean age 20.5 years, *SD* = 2.4, range, 18–28) participated. All participants were native Japanese speakers recruited from the Tohoku University community and all were right-handed, as assessed by the Edinburgh Handedness Inventory [24]. No subject had a history of neurological, psychiatric, or major medical disorders. Written informed consent was obtained from all subjects in accordance with the Declaration of Helsinki [25]. The current study was approved by the Ethics Committee of Tohoku University.

### Stimuli

Each real-life frustrating situation was presented in text form and was followed by presentation a picture taken from the first-person perspective of the participant (Figure 1a, b). Each picture contained a message to the protagonist from the person responsible for the frustrating situation. The participant’s verbal response to the frustrating situation (expressed either to the person responsible for the situation or to one’s self) was then presented as a sentence on the picture (Figure 1c). This sequence was referred to as “a stimulus sequence” (Figure 1). The individual whose photograph appears in the example provided written informed consent for publication of his photograph.

To test the specificity of a significant correlation in adaptive social behaviors, it is necessary to compare adaptive and non-adaptive verbal responses. We referred to stimulus sequences with both adaptive and non-adaptive responses to the same frustrating situation as “a stimulus set” (Figure 2). To prepare the stimulus sets for our acting task, preliminary experiments were conducted in three steps: (1) collection of stimulus sequences including frustrating situations and verbal responses in daily life; (2) categorization of adaptive and non-adaptive responses, which constituted a stimulus set; and (3) verification of the stimulus sets.

In the first step, to elicit honest verbal responses to daily frustrating situations, a separate group of subjects from the Tohoku University community, the same community from which participants in the present study were selected, anonymously completed our original questionnaire asking about daily frustrating situations in their lives as college students and possible verbal responses they would give in such situations. As a result, we identified 41 frustrating situations and seven or eight verbal responses for each situation (a total of 306 verbal responses). Then we obtained 41 pictures, either from the internet or by taking the pictures ourselves, which illustrated 41 frustrating situations.

Seventeen healthy subjects (three women, 14 men; 19–25 years old) were tested separately in the second step. Each subject looked at every stimulus sequence. They were then asked to “imagine being the protagonist in each frustrating situation and evaluate how naturally they would utter a prepared response.” The evaluation of each verbal response was used as a reference for the “causality” scores. To categorize the different types of verbal responses, we performed a discriminant analysis using the SPSS software (ver. 15.0 for Windows; SPSS Inc., Chicago, IL, USA). The causality scores were used as independent variables in the discriminant analysis because the effect of the different verbal responses on the causality scores differed among individuals.

Additionally, we wanted to use an experimental approach to demonstrate that the two factors of adaptation (i.e., “self-performing” and “containing a solution”) were significant explanations for the verbal responses to the frustrating situations. Based on this analysis, verbal responses were divided into four categories by two discriminant functions that accounted for 95.3% of the variance.

The functions were two types of verbal responses to the frustrating situation; these were referred to as performer (self/other-performing) and solution (with/without solution), corresponding to the direction and type of aggression in the Rosenzweig model, respectively [1]. We categorized these two factors into four types of verbal responses (Table 1): self-performing/with solution (SW), self-performing/without solution (SWo), other-performing/with solution (OW), and other-performing/without solution (OWo). In this context, the SW response satisfied the definition of adaptation as a process used to manage environmental demands [3,4] because the SW response does not suggest that a burden will be placed on the environment. As a result of dividing the verbal responses into four categories, the stimulus set contained each frustrating situation, four verbal responses for the conditions of interest (SW, SWo, OW, and OWo; Table 1), and one response as a control condition (Co; Figure 2). Under the Co condition, each subject read the description of the situation itself instead of the verbal responses.

In the third step, a preliminary psychological test conducted with 10 different healthy subjects (five women, five men, 19–29 years old) was used to verify the stimulus set. We eliminated responses understood by fewer than 90% of the subjects. Accordingly, 16 stimulus sets including four types of verbal responses met this criterion (see, Additional file 1, Appendix). The means and standard deviations of the length of the verbal responses were 11.9 ± 1.4, 13.1 ± 1.8, 12.4 ± 1.7, 13.0 ± 2.2, and 13.1 ± 1.4 moras (a prosodic unit of the Japanese language) under the SW, SWo, OW, OWo, and Co conditions, respectively. A one-way analysis of variance (ANOVA) revealed no significant difference in the average length of the responses across conditions [*F*(4,75) = 1.68]. Accordingly, we assumed that behavioral outputs, such as the speech and eye movements required for reading the verbal responses, were similar across conditions.

### Task

Prior to the fMRI experiment, subjects practiced the acting task outside the scanner with four stimulus sets not used in the experiment. The subjects then viewed all the stimulus sequences without verbal responses to ensure that they could understand the frustrating situations.

**Figure 3:**
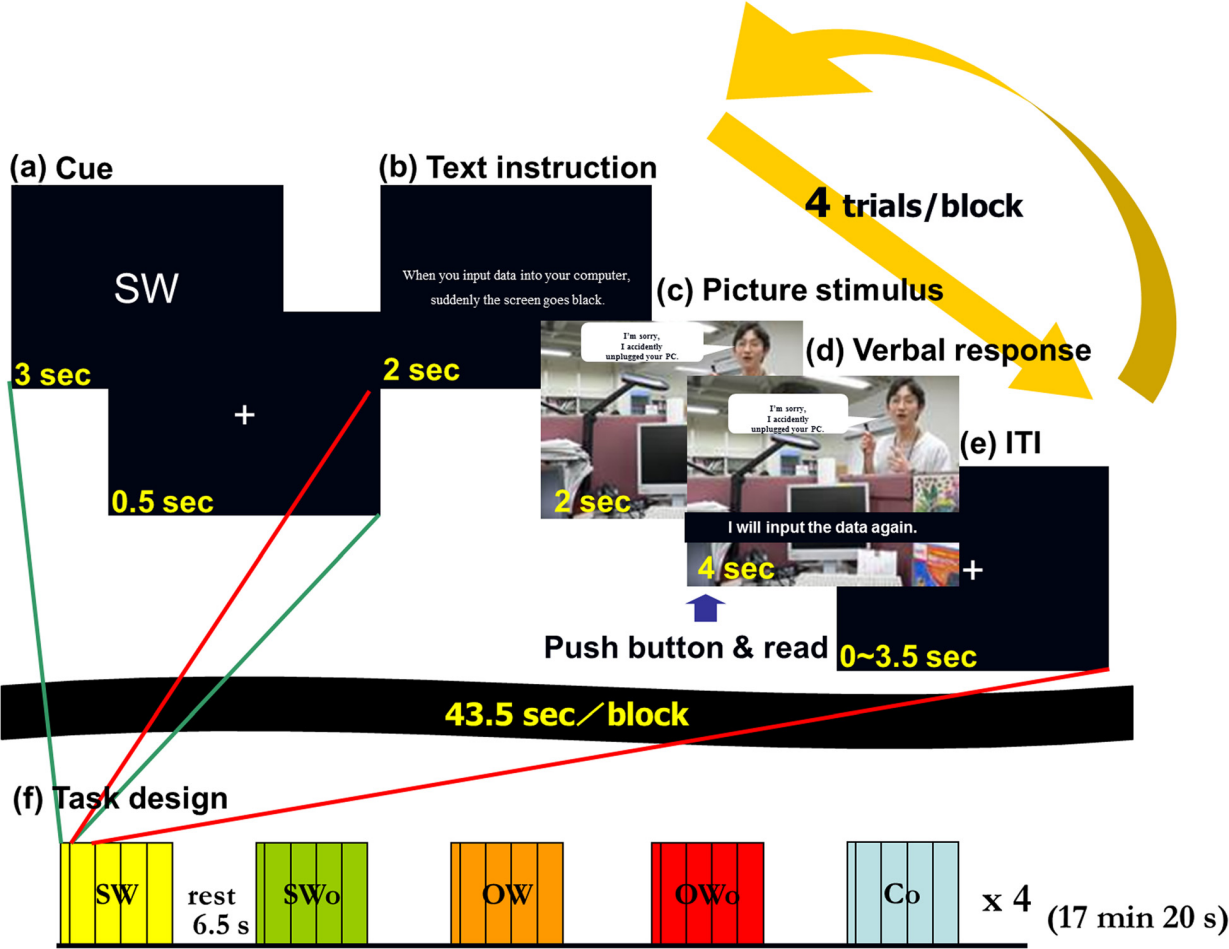
**Task procedure.** (**a**) At the start of each block, a text cue indicating which type of verbal response was to be adopted was presented for 3 s. (**b**) In each trial, a general explanation was presented for 2 s. (**c**) The picture stimulus was then shown for 2 s, and (**d**) the verbal response was elicited for 4 s. The subjects were asked to read the verbal response soon after it appeared and to press a button with their right index finger while reading. (**e**) The intertrial interval (ITI) varied from 0 to 3.5 s. (**f**) Four trials with the same types of verbal responses were presented in one block. The duration of each block was 43.5 s. The interval between blocks was 6.5 s, during which time a central fixation cross (+) was presented to signal the resting condition. Eighty stimuli (16 stimulus sets, 20 blocks) were presented while subjects underwent fMRI scanning; the order of stimuli and blocks were counterbalanced among subjects. The entire scanning time was 17 min, 20 s.

During the fMRI experiment, each subject performed the acting task. Subjects acted as protagonists (i.e., as if they had been in the situation presented in the stimulus picture) by reading a verbal response with feeling. By fixing the headphones between the head coil and the temple areas, head motions were dissociated from jaw movements during reading. To record the entirety of the response, subjects were asked to press a button with their right index fingers while reading the verbal response aloud. A mixed design was applied to this fMRI experiment. To allow a parametric modulation analysis for each trial [26], the inter-trial intervals varied from 0 to 3.5 s, as in an event-related model. Because our primary objective was to have subjects act out the task, we used a block design to decrease the load involved in switching among different types of verbal responses. In brief, four trials using the same type of verbal responses were arranged in a block design. The details of the task procedure are shown in Figure 3.

### Causality scores

After fMRI scanning, the subjects were presented with all of the verbal responses to the frustrating situations outside the scanner. The subjects were not informed of this task prior to scanning. They were asked to evaluate the similarity between the verbal responses given in the scanner and their natural responses to real-life situations using a nine-point scale (1: not at all natural and 9: very natural); these were referred to as causality scores. Subjects were asked to evaluate causality after the fMRI task because an evaluation during scanning may have interfered with their ability to fully immerse themselves in role playing. The causality score was defined as an index of the internal causal attribution of the described response to the hypothetical scenario. Consistent with this definition, when verbalizing a more natural response (i.e., internal causality), the subjects’ causality scores would be higher than when verbalizing a less natural response (i.e., reflecting external causality).

### Psychological measurements

All subjects completed the Japanese version of the STAXI [27] and the Japanese version of the TCI [28]. The STAXI assesses the intensity of feelings of anger (state anger), the disposition to experience anger (trait anger), behaviorally expressed anger (anger-out), suppressed anger (anger-in), and self-control of anger behaviors (anger-control) [23]. The TCI has four independent temperament dimensions (novelty-seeking, harm-avoidance, reward-dependence, and persistence) and three independent character dimensions (self-directedness, cooperativeness, and self-transcendence) [22]. Self-directedness refers to the ability to control one’s own behavior to achieve one’s own goals. Cooperativeness refers to acceptance of others, which induces socially adaptive behaviors such as social tolerance, helpfulness, and compassion. Self-transcendence is related to a kind of spirituality associated with the notion that everything is an essential and consequential part of the universe [22,29].

### fMRI measurement

Transaxial gradient-echo images (number of slices = 44, echo time = 50 ms, flip angle = 90°, slice thickness = 2.2 mm, slice gap = 0.7 mm, FOV = 192 mm, and matrix = 64 × 64) covering the whole cerebrum were acquired at a repetition time of 4000 ms using an echo planar sequence and a Siemens Symphony (1.5 T; Siemens, Erlangen, Germany) MR scanner. To allow for T1 equilibration effects, eight dummy scans were acquired and subsequently discarded. Additionally, anatomical T1-weighted images (thickness, 1 mm; FOV, 256 mm; data matrix, 192 × 224; TR = 1900 ms; TE = 3.93 ms) were acquired from all participants.

### Imaging data analysis

The following preprocessing procedures were performed using the Statistical Parametric Mapping (SPM2) software (Wellcome Department of Imaging Neuroscience, London, UK) and MATLAB (Mathworks, Natick, MA, USA): correction for head motion, adjustment of acquisition-timing across slices, coregistration to the anatomical image, spatial normalization using the anatomical image and the MNI template, and smoothing using a Gaussian kernel with a full-width-at-half-maximum of 10 mm. Data from eight subjects (one woman, seven men) with excessive head motion (more than 2 mm) were excluded. Thus, data from 32 subjects were analyzed.

A conventional two-level approach for fMRI data was adopted using SPM2. We designed two models of expected signal changes for each of the four types of verbal response conditions: canonical signal changes among trials (c: canonical model) and parametrically modulated signal changes correlated with the causality scores within each trial (p: parametric modulation model). The causality scores were normalized to a mean of zero under each condition to orthogonalize the four conditions. Under the Co condition, we designed only a canonical model. A voxel-by-voxel multiple regression analysis of the expected signal changes for each of the nine models (SW-c, SW-p, SWo-c, SWo-p, OW-c, OW-p, OWo-c, OWo-p, and Co) was then applied to the preprocessed images for each subject. The expected signal changes were constructed using the hemodynamic response function provided by SPM2. Parameter estimates from the parametric modulation analysis appeared as the degree of correlation between the causality scores and the signal changes. Statistical inference about the contrast of parameter estimates was performed with the second-level between-subjects (random effect) model using one-sample *t*-tests. The height threshold of all voxel-by-voxel analyses was set at *p* < 0.001, uncorrected, and the extent threshold was set at *p* < 0.05 for multiple comparisons [30,31].

First, to identify the neural networks involved in acting out the virtual frustrating situations, we tested the contrast of the parameter estimates of the canonical model under each of the four conditions compared with the Co condition. The neural responses associated with the adaptive social response were identified by testing the contrast of SW versus each of the other three conditions. Second, to identify the neural responses related to natural and less natural social responses to the SW conditions, we used an event-related model to allow for a parametric modulation analysis of each trial [26] and tested for positive and negative correlations. To exclude the possibility of detecting deactivation correlated with the causality score, we restricted the analyses to the areas in which activation was significant under the SW conditions versus the Co condition (i.e., a mask; p < 0.05, uncorrected). Finally, to examine the specificity of the SW conditions, we performed a paired *t*-test between the SW condition and the three other conditions using parameter estimates of the peak activations of the cluster derived from the parametric modulation analysis.

## Results

### Behavioral results

The means and standard deviations of the durations recorded for the button press while acting out each condition were 2.16 ± 0.25, 2.27 ± 0.25, 2.20 ± 0.26, 2.29 ± 0.24, and 2.31 ± 0.23 s under the SW, SWo, OW, OWo, and Co conditions, respectively (Table 2). No significant difference in duration was observed among the five conditions [one-way ANOVA; *F*(4,155) = 2.10], suggesting that the behavioral output required for reading the verbal responses was controlled across conditions.

**Table 2:** Behavioral data

**Condition**	**SW**	**SWo**	**OW**	**OWo**	**Co**
Duration (s)	2.16 ± 0.25	2.27 ± 0.25	2.20 ± 0.26	2.29 ± 0.24	2.31 ± 0.23
Causality score	5.25 ± 1.19	5.82 ± 0.96	4.72 ± 1.06	6.26 ± 1.30	-

The duration of the acting task is shown for each condition. No significant difference in duration was observed among the five conditions [one-way ANOVA; *F*(4,155) = 2.10]. Causality scores under the four coping conditions are shown. A two-way repeated-measures ANOVA revealed a significant main effect of solution [*F*(1, 31) = 50.47, *p* < 0.05] and a significant interaction [*F*(1, 31) = 12.98, *p* < 0.05]. *Post-hoc* paired t-tests revealed that causality scores under the OWo condition were significantly higher than those under the SW [t(31) = -2.81, p < 0.05, Bonferroni correction] and the OW conditions [t(31) = -8.11, p < 0.0001, Bonferroni correction]. Causality scores under the SWo condition were significantly higher than those under the OW condition [t(31) = 5.39, p < 0.0001, Bonferroni correction]. The values are shown as means and standard deviations. SW, self-performing/with solution; SWo, self-performing/without solution; OW, other-performing/with solution; OWo, other-performing/without solution; Co, control condition.

The mean values of the causality scores for each condition were 5.25 ± 1.19, 5.82 ± 0.96, 4.72 ± 1.06, and 6.26 ± 1.30 under the SW, SWo, OW, and OWo conditions, respectively. The two-way repeated measures ANOVA revealed a significant main effect of solution [*F*(1,31) = 50.47, *p* < 0.05] and a significant interaction [*F*(1,31) = 12.98, *p* < 0.05] (Table 2). *Post hoc* paired *t*-tests revealed that the causality scores under the OWo condition were significantly higher than were those under the SW [*t*(31) = -2.81, *p* < 0.05, Bonferroni correction] and the OW [*t*(31) = -8.11, *p* < 0.0001, Bonferroni correction] conditions, and that those under the SWo condition were significantly higher than were those under the OW condition [*t*(31) = 5.39, *p* < 0.0001, Bonferroni correction].

The correlation analysis between the causality scores in each condition and the psychological questionnaire scales revealed a significant positive correlation between the average causality score in the SW conditions and the Anger Control subscale of the STAXI (*r* = 0.43, *p* = 0.014), as expected. Although we did not find a significant positive correlation between the causality scores in the SW conditions and the C subscale of the TCI (*r* = 0.29, *p* = 0.10), the analysis showed the positive tendency we expected. Furthermore, the causality scores in the SWo conditions had a significant positive correlation with the C subscale of the TCI (*r* = 0.37, *p* = 0.037), whereas the causality scores in the OWo conditions were negatively correlated with the C subscale of the TCI (*r* = -0.41, *p* = 0.020).

### fMRI results

#### Neural correlates of social responses to frustrating situations

**Figure 4:**
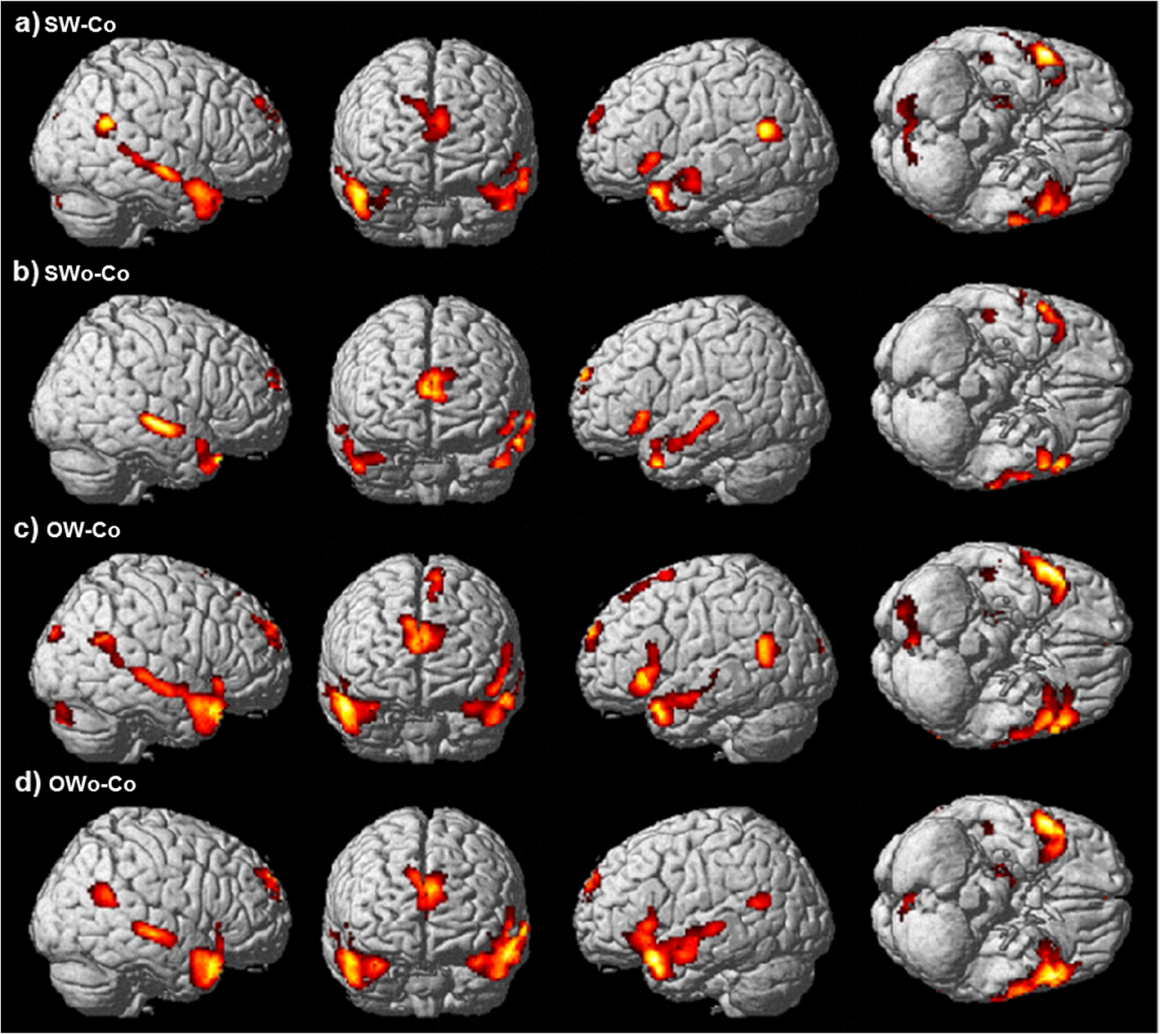
**Neural correlates of social responses in frustrating situations.** High levels of brain activity estimated from a conventional subtraction analysis of the (**a**) SW, (**b**) SWo, (**c**) OW, and (**d**) OWo conditions relative to the Co condition are overlaid on a SPM2 rendered brain. The panels show the right sagittal, anterior, left sagittal, and ventral views of the brain. The statistical threshold was set at *p* < 0.001 and corrected to *p* < 0.05 for multiple comparisons using cluster size.

The brain regions significantly activated in each of the four conditions, relative to the Co condition, are shown in Table 3 and Figure 4. Statistically significant activation under all conditions relative to the Co condition was observed in the medial prefrontal cortices, the left inferior frontal gyrus, the bilateral temporal lobes, and the bilateral occipital lobes. The OW coping style, but not the other conditions, was associated with significant activation of the dorsal part of the medial prefrontal cortices, the supplementary motor area, the right inferior frontal gyrus, and the parietal lobe. Statistically significant activation in all conditions, with the exception of the SWo coping style, was observed in the bilateral temporoparietal junctions, the orbito-insular junction, the bilateral hippocampus/parahippocampus, and the cerebellum. We found no significant differential activation between the SW and the other three conditions.

**Table 3:** Neural correlates of social responses to frustrating situations

**Structure**			**SW–Co**	**SWo–Co**	**OW–Co**	**OWo–Co**
Temporal lobe	Temporal pole	L	−56, 12, 22	−44, 12, –36	−48, 14, –30	−52, 16, –20
			(5.70, 6464a)	(6.05, 3992a)	(6.29, 7344a)	(6.95, 17048a)
		R	54, 16, –32	50, 16, –38	54, 10, –22	50, 14, –36
			(7.81, 10312b)	(5.69, 2304b)	(7.04, 17544b)	(7.19, 10112b)
	Anterior/posterior superior temporal sulcus	L	−46, 12, –36	−62, 0, –22	−60, 10, –18	−58, –4, –18
			(5.45, a)	(4.32, a)	(5.01, a)	(5.86, a)
				−66, –24, –2		−68, –22, –2
				(5.36, a)		(5.75, a)
		R	56, –12, –12	56, –20, –6	56, –12, –10	58, –10, –8
			(7.06, b)	(8.24, 3864c)	(8.09, b)	(7.85, 4200c)
			56, –42, 6		58, –20, –8	48, –26, –6
			(4.50, b)		(6.24, b)	(5.97, c)
	Temporoparietal junction	L	−52, –58, 20		−54, –56, 12	−52, –56, 18
			(6.15, 3880c)		(7.87, 4072c)	(4.21, 1688d)
		R	52, –54, 26		64, –54, 22	60, –54, 22
			(5.59, 2528d)		(5.63, b)	(7.21, 2552e)
	Orbitoinsular junction	L	−26, 12, –16		−28, 14, –18	(*, a)
			(3.83, a)		(4.96, a)	
		R	32, 16, –20		32, 18, –20	30, 12, –22
			(4.08, b)		(4.97, b)	(4.71, b)
	Hippocampus/ parahippocampus	L	−10, –42, –6		(*, d)	
			(5.24, 2656e)			
		R	14, –26, –12		16, –26, –14	14, –28, –14
			(6.07, 2896f)		(5.37, 15096d)	(5.85, 3512f)
Frontal lobe	Superior frontal gyrus	M	−4, 56, 24	4, 56, 18	2, 54, 24	0, 56, 26
			(5.23, 4064 g)	(5.16, 4960d)	(5.91, 7984e)	(6.28, 7584 g)
	Supplementary motor area	M			−6, 10, 64	
					(5.00, 2392f)	
	Inferior frontal gyrus	L	−52, 20, 0	−56, 22, –4	−54, 26, –6	−56, 22, 0
			(5.14, 1936 h)	(5.28, 1592e)	(6.17, 5848 g)	(6.30, a)
		R			50, 24, –8	48, 24, –10
					(4.32, b)	(4.01, b)
Parietal lobe	Precuneus	L			−4, –56, 32	
					(6.30, d)	
		R		8, –56, 36	6, –56, 18	
				(4.16, f)	(4.68, 15096d)	
Occipital lobe		L	−4, –72, 18	−12, –56, 8	−16, –62, 6	−8, –74, 10
			(4.54, 1784i)	(5.15, f)	(6.32, d)	(4.50, 1768 h)
		R	14, –38, –2	20, –62, 6	16, –80, 24	12, –52, 2
			(4.33, f)	(5.20, 2584 g)	(5.41, 5480 h)	(4.95, f)
				18, –78, 26		
				(5.20, 8120f)		
Cerebellum		L	−16, –82, –30		(*, i)	0, –84, –26
			(4.47, j)			(5.53, 1568i)
		R	10, –78, –30		24, –80, –36	8, –78, –18
					(5.93, 3944i)	(5.12, i)
			(4.87, 3224j)			

MNI coordinates (*x*, *y*, *z*) of peak activation, *t*-value at the peak, and cluster size (mm^3^) in parenthesis are given for each area activated by each coping style, relative to the Co condition. The height threshold for significant activation was set at *p* < 0.001. Correction for multiple comparisons (*p* < 0.05 in cluster size) was made. L: left, R: right, M: medial. The lowercase letter given with the cluster size indicates that the peak is in the same activated cluster as are the other peaks with the same letter. (*) The region includes the activated cluster with no peak activation.

### Neural correlates of natural adaptive social responses to frustrating situations

The parametric modulation analysis showed no significant positive correlations between the causality scores and neural responses in any condition.

### Neural correlates of less natural adaptive social responses to frustrating situations

**Figure 5:**
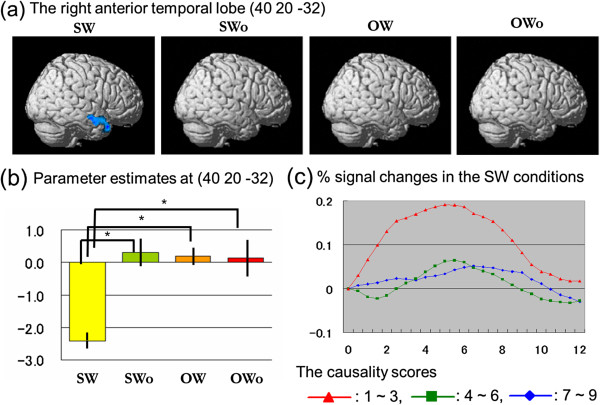
**Neural correlates of less natural adaptive social responses.** (**a**) The right anterior temporal lobe was activated only under the SW condition, as shown on the rendered brain presentation of the SPM2 from the right sagittal view. No significant correlation was found between neural activation and the causality score under the SWo, OW, or OWo conditions. The statistical threshold was set at *p* < 0.001 and corrected to *p* < 0.05 for multiple comparisons using cluster size. (**b**) The graphs show activation profiles (parameter estimates from a parametric modulation analysis and standard errors of the mean at peak activation). *p < 0.05, paired *t*-test. (**c**) The average percent signal changes over time are shown for trials with low (1–3; red triangles), medium (4–6; green squares), and high (7–9; blue diamonds) causality scores under the SW condition. Vertical axes represent percent signal changes; horizontal axes indicate peristimulus time (s).

The parametric modulation analysis revealed a significant negative correlation only between the SW condition causality scores and neural responses in the right anterior temporal lobe (Table 4, Figure 5a). The parameter estimates showed significant differences between the SW condition and the other three conditions at the peak voxel in this cluster (Table 5, Figure 5b). The time-series data for the percentage signal change at the peak statistical value in this area showed larger responses for the low-causality scores compared with the medium- and high-causality scores (Figure 5c).

**Table 4:** Neural correlates of less natural adaptive social responses to frustrating situations

**Structure**	**MNI coordinate**			***t***	**Cluster size**
	***x***	***y***	***z***		**(mm**^**3**^**)**
Right temporal pole	40	20	−32	5.04	3768
Right anterior superior temporal sulcus	64	−8	−18	4.72	

The results of the significant negative correlation between the agreement scores and neural responses for the SW verbal responses masked by the SW–Co contrast inclusively. The coordinates, *t*-value of peak activation, and cluster size are shown for each activated area. The height threshold for significant activation was set at *p* < 0.001, and corrected to *p* < 0.05 for multiple comparisons using cluster size.

**Table 5:** Parameter estimates at the anterior temporal lobe [40 20 -32]

**Condition**	**SW**	**SWo**	**OW**	**OWo**
Parameter estimates	−2.41 ± 2.71	0.31 ± 4.83	0.20 ± 2.93	0.13 ± 6.25

A significant difference was observed among the four conditions [one-way ANOVA; *F*(3,124) = 2.81, *p* = 0.41]. Significant differences between SW and the other three conditions were detected using a paired *t*-test (*t*(31) = –2.68, *p* = 0.012; *t*(31) = –3.62, *p* = 0.001; *t*(31) = –2.51, *p* = 0.017, respectively.

## Discussion

The aim of the present study was to identify the neural correlates of adaptive social responses to frustrating situations by assessing causal attributions. We found a significant negative correlation between causality scores and brain activity in the right anterior temporal lobe while acting out adaptive social responses. The negative correlation indicated a less natural social response, suggesting that this region is specifically activated when an adaptive social response is driven by an external causal attribution.

### Rosenzweig model and social adaptation

We revealed the neural correlates of the specificity of social adaptation based on the Rosenzweig model. In the current experiment, we combined the Rosenzweig model for social responses to frustrating situations [1,2] and social adaptation, proposed according to coping theory [3]. Through preliminary experiments, we identified two factors that contribute to social responses to frustrating situations, consistent with the Rosenzweig model. However, although the Rosenzweig model is a 3 × 3 factorial design (i.e., direction of aggression involving self, another person, and nothing × type of aggression, involving attention to the frustrating event itself, to the cause of the frustrating situation, and a to solution to the frustrating situation) [1,2], social responses in our results consisted of a 2 × 2 factorial design (i.e., self/other performing × containing/not containing a solution). Logical thought requires that social responses corresponding to self-/other-performing are consistent with those directing aggression toward the self and another person, respectively. However, those corresponding to the direction of aggression involving requests toward nothing were excluded from our stimuli through the discriminant analysis. The number of responses that were requests toward nothing was not sufficient to create a significant category. For the type-of-aggression factor, we could regard those with attention to the frustrating event itself and to a cause of the frustrating situation as “no solution” and regard a solution to the frustrating situation as containing a solution. Although a 3 × 3 factorial design was integrated into a 2 × 2 design, at least two factors of the Rosenzweig model were replicated in the present study.

Moreover, we found specific neural responses under SW conditions that were related to socially adaptive responses [3]. In the current study, we focused on the internal (i.e., self-performing) and external (i.e., containing a solution that implicitly responds to external demands) causality of social responses. Adaptive social responses may have a stronger association with external causal attributions than do non-adaptive social responses because the definition of social adaptation [3] refers to managing environmental demands to solve problems, which corresponds to external causality. Although other definitions of adaptation have been proposed [32,33], the one developed by Lazarus [3] was congruent with respect to specificity. Therefore, our data showing that a specific neural response was elicited only when adaptive behavior was observed support the appropriateness of the definition proposed by Lazarus [3] for use in efforts examining the biological underpinnings of socially adaptive responses.

### Causality scores and psychological measurements

We found a significant correlation between causality scores and responses to the psychological questionnaires. This finding suggests that the internal causal attribution of adaptive social responses (i.e., the SW condition) requires self-control and the ability to control anger behavior in frustrating situations. Moreover, self-performing responses (i.e., the SW and SWo conditions), which put no burden on others, were associated with a cooperative personality. This is in contrast to the OWo condition, which represents a complaining/accusing attitude and is associated with a less cooperative personality. These findings reinforce the validity of the causality scores, as we predicted.

On the other hand, we observed an apparent discrepancy between the behavioral and fMRI results regarding causality scores. Although we found a significant main effect of solution and a significant interaction in the causality scores among the four conditions, we did not detect a significant main effect or interaction among the parameter estimates derived from the parametric modulation analysis. The results of the parametric modulation model depend on within-subject variation in the causality scores under each condition, whereas the mean values of the causality scores reflect individual variability across subjects. Thus, the result related to the specificity of parameter estimates under the SW condition was robust.

### Right anterior temporal lobe

Although previous neuroimaging studies have not reported right anterior temporal lobe involvement in causal attributions, the cognitive function of this region highlights the importance of integrated processing for adaptive social behavior. A previous review suggested that the lateral prefrontal cortices contributed to adaptive social reasoning because they had been shown to mediate cognitive functions, such as social exchange, simulation, integration, deductive and inductive inference, and social cognition [15]. One interpretation of our results is that adaptive social behaviors with an external causal attribution (i.e., less natural social responses) require a high cognitive load for the integration of emotional and social information. Previous studies have shown right anterior temporal lobe involvement in cognitive integration in social contexts. For example, this region was activated during the bottom-up processing of breaches occurring in social contexts, such as social norm violations [18] and social–cognitive conflicts [34]. Additionally, this region is involved in the top-down processing of comprehension in a social context, such as moral reasoning [14,16], mentalizing (understanding others’ intentions) [35], irony [36], and abstract conceptual knowledge [37]. Olson et al. argued that the right anterior temporal lobe was involved in the emotional processing associated with social relationships (socio–emotional processing) [38]. Other reports have suggested that the functional role of the anterior temporal lobe can be understood in terms of its serving as a semantic hub mediating social–emotional processing [39]. In addition to these cognitive processes, the region plays a role in behavioral processes, such as producing communicative speech in a social context [40]. Thus, neural activation in this region integrates social stimuli involving the social demands of frustrating situations. Such social demands constitute the characteristics of external causal attribution, particularly in the context of an adaptive social behavior. The functional role of this region highlights the importance of integration in the performance of adaptive social behaviors.

### Neural networks for social responses to frustrating situations

The neural networks associated with social responses to frustrating situations identified in the present study are consistent with those previously reported in neuroimaging studies investigating social behaviors. These brain areas are part of the brain network that mediates social cognition and behavior [41,42], such as mentalizing [35], social interaction [43], and communicative speech production [40]. Furthermore, they are related to judgments about the adaptiveness of behavior in relation to a social context with regard to considerations such as social norms [18,44] and moral judgments [14,16,45]. A comparison of our results with previous findings suggests that subjects must understand the meaning of a social situation to produce communicative speech during the acting task, and that this may be related to environmental demands.

We failed to find neural correlates of adaptive social responses via conventional subtraction analyses (i.e., contrast between adaptive and non-adaptive responses). On the other hand, after fitting the causality scores, we were able to detect adaptive-specific neural responses. The results indicate that the concept of causality is needed in attempts to identify the neural correlates of adaptive-specific neural responses under experimental conditions.

### Methodological considerations and future studies

Certain methodological issues in our experiment must be considered. First, the validity of our novel methodology was supported by both psychobehavioral and fMRI results. The correlation between the causality scores and the psychological measures reinforce the validity of the causality scores. Moreover, the significant correlation between neural activation and the causality scores, together with the role of the right anterior temporal lobe in cognitive function, enabled us to interpret the fMRI results and support the validity of our novel methodology.

Second, the reason we failed to find a significant positive correlation between brain activity and causality scores in any experimental condition should be considered. We believe this was the result of the definition we used for adaptation and the characteristics of our acting task. The definition of adaptiveness, which focused on environmental demands corresponding to an external causal attribution, was taken from Lazarus’ 1984 article [3]. Furthermore, our acting task was designed to emphasize a frustrating situation corresponding to external causal attributions. Thus, the subjects were likely to process an external causal attribution, derived as a negative correlation between brain activation and the causality scores, more explicitly. Separate studies optimized to highlight internal causal attribution are necessary to address natural adaptive social behaviors. To accomplish this, an alternative definition of adaptiveness, focusing on the processes used to manage environmental and internal demands [32,33], must be developed. Consistent with this definition [32,33], the internal demands were related to real-life adaptive social behaviors, which could be derived as a positive correlation.

The final issue is gender differences in the adaptive social responses to frustrating situations. Although a previous study reported a gender difference in the response to social stimuli [46], we did not examine gender differences in adaptive social behaviors. We were only able to recruit eight female subjects for the present study and found no significant differences in this subsample. We assessed the issue of mixed data from all male and female subjects and found that an analysis of the results from the male subjects alone showed the same tendency as did the results for all subjects. Thus, we believe the results are valid despite the small number of female participants.

## Conclusions

This is the first reported study to investigate the neural correlates of adaptive social behaviors by assessing causal attributions using hypothetical scenarios under experimental conditions. We believe this novel approach has the potential to open up entirely new areas of research using neuroimaging methods. In particular, we believe that the recent development of a visual cortex decoding device using neuroimaging techniques gives neuroimaging studies the potential to provide objective assessment tools for clinical settings.

## Competing interests

We have no conflicts of interest to declare.

## Authors’ contributions

All authors contributed to the concept and design of the present study. AS and MS contributed to data acquisition. AS, MS, SY, YS, and RK contributed to data analysis and interpretation. AS, MS, SY, YS, and RK provided statistical expertise. AS was the primary writer of the manuscript. MS, YT, YS, and RK were the primary reviewers/revisers of the manuscript. All authors discussed the results and commented on the manuscript. All authors gave their final approval for submission of the manuscript.

## Supplementary Material

Additional file 1 List of stimuli sets. Click here for file
