# Supplementary material for: Neural correlates of adaptive social responses to real-life frustrating situations: a functional MRI study
Source: BMC Neurosci. 2013 Mar 13;14:29. doi: 10.1186/1471-2202-14-29 (PMC3605341; doi:10.1186/1471-2202-14-29)
Supplement: Additional file 1 — List of stimuli sets. [file 1471-2202-14-29-S1.docx]

Appendix: List of stimuli sets

| Frustrating situations explained using text and pictures. | Condition | Verbal response |
| --- | --- | --- |
| 1. Although it was a short distance to the destination, I take a taxi. The taxi driver says, “You can walk there.” | SW | I will walk there. |
|  | SWo | I should have taken a bus. |
|  | OW | Please take me there. |
|  | OWo | You shouldn’t have said that. |
| 1. After I am covered by mud splashed by a car on the road, the driver says, “I am sorry for splashing the mud.” | SW | I will send it to the laundry. |
|  | SWo | I shouldn’t walk on this road. |
|  | OW | You should get off a car first. |
|  | OWo | Why can’t you be careful? |
| 1. When I am 5 minutes late for a part-time job, the boss says, “Do you know what time it is?” | SW | I’m sorry, I'll be careful from now on. |
|  | SWo | I shouldn’t have taken a part-time job. |
|  | OW | Tell me what I should do now. |
|  | OWo | You shouldn’t get mad, it’s only 5 minutes. |
| 1. Although I want to go to the sixth floor by elevator, I push the button for the fifth floor by mistake. When the elevator stops at the fifth floor, a lady on the elevator says, “Is anyone getting off on the fifth floor?” | SW | Thank you for asking. |
|  | SWo | I pushed the button for the fifth floor by mistake. |
|  | OW | Please close the door. |
|  | OWo | You should have noticed without asking. |
| 1. I treat my assistant to sushi. Although he eats a lot of sushi, he says, “May I order more?” | SW | You may order and eat more. |
|  | SWo | I shouldn’t have treated him. |
|  | OW | You should make sure you’re hungry |
|  | OWo | You shouldn’t eat so much. |
| 1. At the cash register of an expensive restaurant my friend says “I don’t have any money on me, please pay the bill.” | SW | I will pay the bill |
|  | SWo | I should have chosen a less expensive restaurant. |
|  | OW | I will wait while you go withdraw money. |
|  | OWo | You should have brought enough money. |
| 1. At the cash register of a restaurant at which I planned to use a coupon for a 30% discount, the cashier says, “You can’t use this coupon today.” | SW | I will pay with a credit card. |
|  | SWo | I should have confirmed it in advance. |
|  | OW | Call the store manager. |
|  | OWo | You shouldn’t have made such a confusing coupon. |
| 1. I have eaten the last piece of cake in the refrigerator. My colleague says, “You should have left one for me.” | SW | I will buy another cake. |
|  | SWo | I shouldn’t have eaten it. |
|  | OW | You can buy another cake. |
|  | OWo | You should have told me you wanted cake. |
| 1. When I try to join a sports club, the receptionist says, "Students cannot register as club members." | SW | I will join after I graduate. |
|  | SWo | I should have checked that. |
|  | OW | Will you let me register as a member if I pay the fee? |
|  | OWo | Why can’t students become members? |
| 1. I confidently ask a question at the conference, but the chairman says, “Don’t you understand such a simple concept?” | SW | I will find the answer myself. |
|  | SWo | It was a stupid question, wasn’t it? |
|  | OW | Can I get an answer soon? |
|  | OWo | You shouldn’t have said that. |
| 1. When I get to the early morning lecture, "Today’s lecture is canceled" is written on the blackboard. | SW | No way! I will take a nap here. |
|  | SWo | I should have checked the bulletin board. |
|  | OW | Is there someone who has nothing to do with? |
|  | OWo | The teacher should have notified us. |
| 1. When I try to get a ride in a friend's car, he says, "I have lost the car keys." | SW | I will ask another friend. |
|  | SWo | I should have said I would walk. |
|  | OW | You should look for the keys. |
|  | OWo | You should have checked to see that you have them. |
| 1. While walking down a narrow hallway, I bump into a stranger, and he says, “Shit! Look out.” | SW | I’m sorry. I will walk closer to the wall. |
|  | SWo | I shouldn’t have walked down this narrow hallway. |
|  | OW | You should give way. |
|  | OWo | You shouldn’t have said such a thing. |
| 1. When I take out burnable garbage, a neighbor says, "Today is a non-burnable trash day." | SW | I will take out non-burnable garbage. |
|  | SWo | I should have checked it. |
|  | OW | When is the next burnable trash day? |
|  | OWo | No one notified me. |
| 1. After waiting for 2 hours at the hospital, I ask the receptionist, “How much longer do I have to wait?” She says, “You must wait a while because an emergency patient has come in.” | SW | I will wait for a while. |
|  | SWo | I should have gone to another hospital. |
|  | OW | Please examine me soon. |
|  | OWo | Do you know how long I have been waiting? |
| 1. A friend returns a magazine that I have lent to him and says, “I’m sorry, my younger brother has torn this.” | SW | OK, I will buy a new one. |
|  | SWo | I shouldn’t have lent it to him. |
|  | OW | You should buy a new one. |
|  | OWo | You shouldn’t have let your brother see it. |

In the actual acting task, subjects viewed the text and pictures explaining the frustrating situations. The original version was written in Japanese.
